# Expression of Serum Exosomal miRNA 122 and Lipoprotein Levels in Dogs Naturally Infected by *Leishmania infantum*: A Preliminary Study

**DOI:** 10.3390/ani10010100

**Published:** 2020-01-08

**Authors:** Antonio Di Loria, Vincenzo Dattilo, Domenico Santoro, Jacopo Guccione, Adriana De Luca, Paolo Ciaramella, Marinella Pirozzi, Enrico Iaccino

**Affiliations:** 1 Department of Veterinary Medicine and Animal Productions, University Federico II, 80130 Napoli, Italy; jacopo.guccione@unina.it (J.G.); paociara@unina.it (P.C.); 2 Department of Health Sciences, Magna Graecia University, 88100 Catanzaro, Italy; dattilo@unicz.it; 3 Department of Small Animal Clinical Sciences, College of Veterinary Medicine, University of Florida, Gainesville, FL 32610, USA; 4 Institute of Protein Biochemistry, National Research Council, 88100 Napoli, Italy; marinella.pirozzi@ibbc.cnr.it; 5 Department of Experimental and Clinical Medicine Magna Graecia University, 88100 Catanzaro, Italy; iaccino@unicz.it

**Keywords:** canine leishmaniasis, microRNA, miR-122, lipoprotein, exosomes

## Abstract

**Simple Summary:**

The immunopathogenesis of leishmaniasis is not completely understood. Exosomes are extracellular vesicles produced by most eukaryotic cells, containing various molecular constituents with biological effects (e.g., proteins, peptides, RNA). They play an important role in cell-to-cell signaling. Recently, exosomal microRNA were demonstrated to be able to regulate gene expression and protein production in mammalian cells, serving as potential biomarkers of disease. The microRNA miR-122 is a biomarker of hepatic damage widely studied in mice in the course of *Leishmania* infection. *Leishmania* organisms can interfere with miR-122 production leading to a dysfunction in cholesterol metabolism ensuring its proliferation in the infected host. In this study, we suggest that such a phenomenon may also occur in dogs affected by *Leishmania* infection.

**Abstract:**

Current knowledge on the role of exosomal microRNA (miRNA) in canine leishmaniasis (CL), with particular regards to the interaction between miR-122 and lipid alterations, is limited. The aim of this study was to isolate/characterize exosomes in canine serum and evaluate the expression of miR-122 in ten healthy and ten leishmaniotic dogs. Serum exosomes were isolated using a polymer-based kit, ExoQuick^®^ and characterized by flow cytometry and transmission electron microscopy, whereas miR-122-5p expression was evaluated by quantitative reverse-transcriptase polymerase chain reaction. A significant decreased expression of exosomal miR-122-5p, decreased serum levels of high-density lipoproteins, and increased serum levels of low-density lipoproteins were seen in leishmaniotic dogs when compared with healthy dogs. These results suggest that hepatic dysfunctions induced by the parasite interfere with lipoprotein status. The decreased expression of exosomal miR122 represents an additional effect of *Leishmania* infection in dogs as in people.

## 1. Introduction

Leishmaniasis is a zoonosis caused by intracellular protozoa of the genus *Leishmania* transmitted by phlebotomines. During the initial phase of the infection, *Leishmania* spp. can survive within the Kupffer cells without affecting the hepatic parenchyma [1]. A high tolerability of such cells to *Leishmania* spp. promotes a parasite survival in the canine liver leading to a perturbation of liver function and, in particular, cholesterol and lipoprotein metabolism [2,3]. In fact, *Leishmania* parasites are able to modulate the expression of genes associated with cholesterol biosynthesis, uptake, and efflux [2,4]. Cholesterol plays an important role in *Leishmania* infection since amastigotes are not able to synthesize it de novo [5], however, the mechanistic links between *Leishmania* infection and lipid changes are complex, multifactorial, and not completely understood. Important differences between promastigotes and amastigotes of *Leshmania chagasi* have been observed regarding uptake through lipid rafts, subdomains of the plasma membrane that contain high concentrations of cholesterol and glycosphingolipids. A transient disruption of lipid rafts in cell membranes affected promastigote uptake, but not amastigote uptake by macrophages. These findings indicate a difference in the needs of *Leishmania* parasites regarding both the availability and origin of cholesterol. Leishmania protozoa can alter the metabolism of cholesterol directly or through the effect on lipoproteins; trypanosomatids are able to acquire cholesterol from low-density lipoproteins (LDLs) and high-density lipoproteins (HDLs) by endocytosis [6,7,8].

As in people, Ghosh et al. [9] showed that an inverse association between blood levels of cholesterol and susceptibility to *Leishmania donovani* infection was present in mice. Contrarily, in leishmaniotic dogs, while hyper/normal cholesterolemia has been detected, high levels of low-density lipoproteins (LDLs) and low levels of high-density lipoproteins (HDLs) have been reported [10,11,12].

Recently, microRNAs (miRNAs) have been used to investigate both lipid metabolism and function in animals [13]. miRNAs are small, 20–22 nucleotides long, posttranscriptional regulators identified in tissues and blood in healthy and diseased people and dogs [14,15]. They act on mRNA primarily as inhibitors (translational repression or degradation) affecting several physiological processes [13]. While in circulation, serum miRNAs are highly degradable, however, when transported in microvesicles (exosomes) these molecules are more stable and can serve as reliable diagnostic biomarkers in diseased patients [16,17,18]. Exosomes being small extracellular mycelial vesicles [19] protect RNA from RNAse degradation [20]. In 2013, Ghosh et al. [21] explored, for the first time, the role played by exosomes in miR-122 expression, the most common miRNA present in the liver tissue, in *L. donovani* infection in mice. The authors showed that, the glycoprotein gp63, present in *Leishmania* exosomes, was able to degrade Dicer1 in the hosts’ hepatic cells, reducing the synthesis of miR-122. Considering these premises, the aim of this study was twofold: evaluate the expression of serum exosomal miR-122 and the lipoprotein profile in dogs naturally infected by *Leishmania infantum*.

## 2. Materials and Methods

### 2.1. Animals

Ten mixed breed dogs, naturally infected by *L. infantum*, and ten mixed breed healthy dogs were recruited in the present study. The diagnosis of CL was based on compatible clinical signs and confirmed by visualization of amastigotes in lymph nodal aspirates and serologically by a positive indirect fluorescent antibody test (IFAT) greater than 1:160 [22,23]. All dogs were also tested for presence of *Dirofilaria immitis*, *Anaplasma phagocytophylum, Borrelia burgdorferi*, and *Ehrlichia canis* antibodies using SNAP^®^ test (Canine SNAP 4Dx, IDEXX laboratories). In order to be enrolled, the dogs with leishmaniasis had to be untreated at the moment of diagnosis and negative to the SNAP test. The healthy dogs had to be clinically healthy, negative to IFAT (<1:40) [22,23] and the SNAP test.

### 2.2. Samples Collection and Hemato-Biochemical Analysis

Ten mL of peripheral blood were collected from the jugular vein of each dog and put into tubes without anticoagulant (5 mL) and in tubes containing ethylene diamine tetraacetic acid (EDTA) (5 mL). A complete blood cell count was performed within 30 min from the collection using a semi-automatic cell counter (Genius S; SEAC Radom Group, Florence, Italy). Serum was also collected after centrifugation at 327× *g* for 15 min and it was stored at −20 °C. Serum urea, creatinine, aspartate aminotransferase (AST), alanine aminotransferase (ALT), bilirubin, alkaline phosphatase (ALP), and total protein (TP) were analyzed using commercially available kits (Reactivos Spinreact S.A. OLOT, Gerona, Spain). Total serum cholesterol, triglycerides, and high-density lipoprotein cholesterol (HDL) were measured using a Dimension EXL analyzer (Siemens Healthcare Diagnostics s.r.l., Milan, Italy); low-density lipoprotein cholesterol (LDL) was calculated using the Friedewald equation [24].

### 2.3. Exosomes Isolation and Mirna Detection

Exosomes were extracted from the serum using a polymer-based kit, ExoQuick^®^ (System Biosciences Mountain View, Palo Alto, CA, USA) according to a previous study [17]. Exosomes were analyzed by flow cytometry (FC) and characterized by transmission electron microscopy (TEM). Dynamic light scattering and zeta potential determinations were also performed with a Nano ZS 90 (Appendix A).

Isolated exosomes were processed for miRNA isolation using a commercially available kit (exoRNeasy Serum Plasma Kit; Qiagen, Hilden, Germany). Subsequently, the cDNA was amplified by quantitative reverse-transcriptase polymerase chain reaction (qRT-PCR) following the manufacturer’s instructions (Appendix A).

### 2.4. Statistical Analysis

The data were tested for normal distribution using the Kolmogorov–Smirnov test (alpha = 0.05). The unpaired two samples Student’s *t*-test or Mann–Whitney test was performed to evaluate the behavior of each data variable between the two groups (healthy vs. CL). All statistical comparisons were performed using the GraphPad Prism6 Software (GraphPad Software Inc., La Jolla, CA, USA). A *p* < 0.05 was considered statistically significant.

## 3. Results

### 3.1. Clinical Examination and Blood Tests

The median age at the moment of enrollment was four years (range: 1–6) for the healthy group and four years (range: 1–8) for the CL group. The mean body weight was 22.3 ± 5.4 kg and 20.4 ± 4.3 kg for healthy and CL group, respectively. There were four males and six females (three spayed) in the healthy group, whereas five males (one castrated) and five females (two spayed) were present in the CL group. There were no differences in age (Mann–Whitney; *p* = 0.37), weight (*t*-test; *p* = 0.39), or sex (Fisher’s exact; *p* = 1) between the two groups. The more frequent clinical signs observed in the CL group were lymphadenopathy (80%), weight loss (70%), skin lesions (70%), and splenomegaly (30%). The skin lesions included seborrhea sicca (5) and alopecia (2). The results of hematological and biochemical tests are presented in Table 1.

In particular, 70% of affected dogs had a non-regenerative normocytic normochromic anemia. In addition, CL dogs had a significant reduction in total red blood cells (*p* = 0.01), hematocrit (*p* = 0.0009), hemoglobin (*p* = 0.0001), mean corpuscular volume (MCV, *p* = 0.008), mean corpuscular hemoglobin (MCH, *p* < 0.0001), and mean corpuscular hemoglobin concentration (MCHC, *p* < 0.0001). The biochemical parameters were also altered in the CL group compared to the healthy dogs. In particular, levels of TPs (*p* = 0.0003) and LDLs (*p* = 0.01) were significantly increased, whereas the level of HDLs was significantly decreased (*p* < 0.0001).

### 3.2. Exosomes Isolation and miRNA Detection

**Figure 1:**
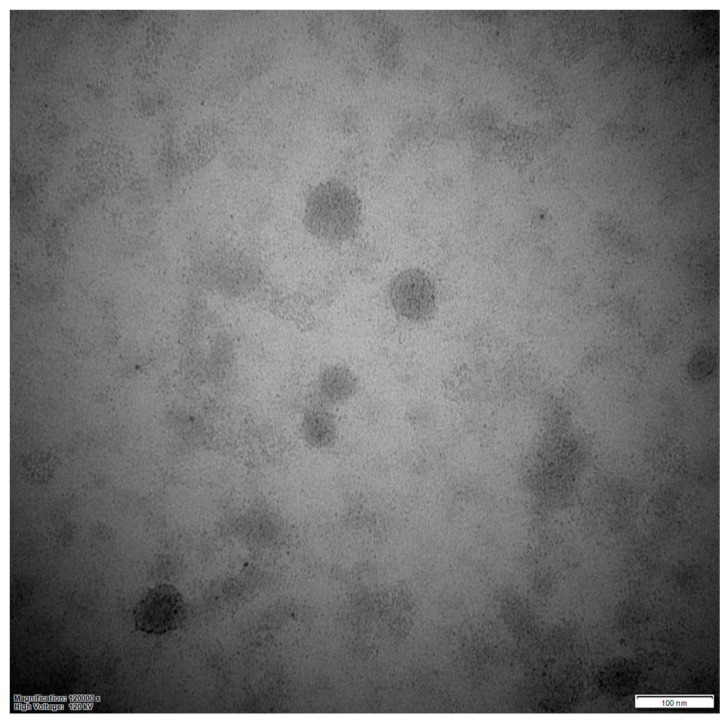
Transmission electron micrograph of exosomes isolated from serum. The morphology is observed by negative staining.

Serum exosomes were detected as round vesicles of heterogeneous sizes via negative stain observed by TEM (Figure 1).

**Figure 2:**
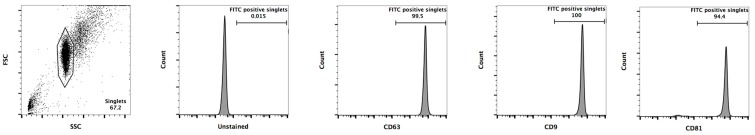
Identification of canine exosomes. Exosomes, obtained from canine serum, were labeled with Exo-FITC and incubated respectively with anti-CD63, anti-CD9 and anti-CD81 Exo-Flow FACS magnetic beads. The data percentage of captured exosomes is shown.

The size determination was further investigated using a Zetasizer Nano resolved in an average size of 131 ± 4 nm with a Z potential of −27 ± 0.5 mV. Fluorescein isothiocyanate (FITC) positive singlets were 99.5%, 100%, and 94.4% for CD63, CD9, and CD81, respectively (Figure 2).

**Figure 3:**
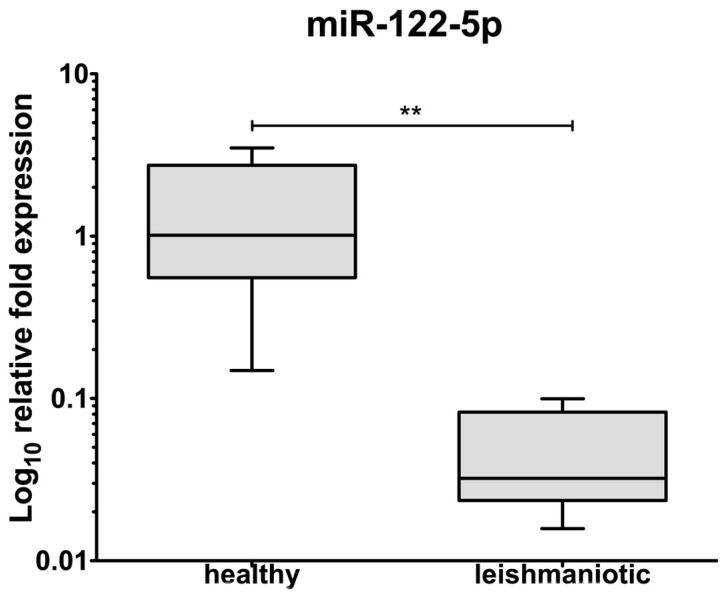
Expression of miR-122 gene expression in canine serum exosomes from healthy (*n* = 10) and leishmaniotic (*n* = 10) dogs. Boxes and whiskers graph. The boxes indicate the quartiles and the mean. **: *p* < 0.01.

A total of 12 ng/μL of miRNA was isolated from serum exosomes. Using qRT-PCR, both miR-122 and RNU6-2, with Ct values of 35.3 ± 0.4 and 32.5 ± 0.6 respectively, were detected. When exosomal miR122 levels were compared between healthy and leishmaniotic dogs, a significantly lower (*p* = 0.004) expression was seen in the latter group (Figure 3).

## 4. Discussion

Although in the present study, a significant modification of the level of serum cholesterol was not present, a significant alteration of serum LDL and HDL levels were seen in the CL group, in agreement with previous studies [10,11,12,21]. Such alterations may suggest a lipid perturbation associated with *Leishmania* infection. These data are also in agreement with Carvalho et al. [28] showing that people with clinical manifestation of visceral leishmaniasis have high triacylglycerol and very-low-density lipoprotein (VLDL) levels, but low HDL levels. Different mechanisms may be implicated in the reduction of HDL levels during *Leishmania* infection; these may include decreased hepatic synthesis and secretion of apolipoproteins [29], increased endothelial lipase activity [30], and displacement of apoA-I by serum amyloid A [31]. In addition to their primary role in lipid transport, HDLs have also been associated with anti-inflammatory and anti-oxidant activity, vascular endothelial cell activation, nitric oxide (NO) production, expression of inflammatory mediators, and endothelial progenitor cell proliferation [32,33,34,35]. A reduction of HDL levels could represent a mechanism of defense that the protozoa uses to contrast the leishmanicidal activity of NO in infected macrophages [36]. In a recent study, Rodrigues Santos et al. [37] showed that human monocytes, experimentally infected by *L. infantum*, had two times higher parasitism in the presence of VLDL and HDL than when these lipoproteins were absent.

This is the first study in leishmaniotic dogs showing higher levels of serum exosomal miR-122, a microRNA recently indicated as a good candidate marker for liver diseases in the absence of liver-specific biochemical markers [15]. In leishmaniotic dogs, liver damage can be present with or without specific clinical signs as well as with a low or high parasitic burden. Indeed, liver granulomas (effector T cells, macrophage/dendritic cell) have been described in asymptomatic dogs with low parasite burdens while not organized granulomas were detected in the liver of symptomatic dogs with high parasite burdens [38]. In this study, liver biopsies were not performed (absence of increased cytopathic markers of liver toxicity, e.g., ALT), however, a lower level of albumin and exosomal miR-122 in the absence of renal and enteric signs, suggests liver dysfunction rather than liver damage. This dysfunction associated with a reduction of circulating miR-122 in leishmaniotic dogs may lead to the hypothesis that, like in mice [21], *Leishmania* parasites may play a potential role in the regulation of specific miRNA through the gp63 in dogs.

Future research should consider enrolling a significantly higher number of dogs naturally infected by *L. infantum* in order to study the connection of exosomal miR-122 obtained both from serum and from liver biopsies with the levels of circulant gp63.

## 5. Conclusions

In summary, the results of the present study suggest that alterations of the lipid metabolism, low HDL and high LDL serum levels along with a lower miR-122 expression may indirectly mirror hepatic alterations induced by *L. infantum* in dogs. However, because of the low number of animals enrolled, further studies are warranted to better define the role of miR-122 as a potential biomarker of hepatic damage/disfunction during canine leishmaniasis.

## Figures and Tables

**Table 1:** Hematological and biochemical parameters in healthy and affected dogs.

Parameter	Healthy	Leishmaniotic	Reference Range [25,26,27]	*p*-Value
RBC (×10^6^/dL)	6.5 ± 0.6	5.5 ± 1.6	5.5–8.5	0.01
Hb (g/dL)	14.9 ± 1.5	10.7 ± 3.1	12–18	0.0001
Hct (%)	44.3 ± 3.2	34.4 ± 9.6	37–55	0.0009
MCV (fL)	69.2 ± 3.4	63.4 ± 4.8	60–77	0.008
MCH (pg)	21.1 ± 1.4	19.6 ± 1.4	20.5–24.2	<0.0001
MCHC (%)	33.8 ± 1.1	30.9 ± 0.9	32–36	<0.0001
PLT (×10^3^/mm^3^)	295.4 ± 89.5	279.3 ± 74.4	200–500	0.7
WBC (×10^3^/mm^3^)	10.3 ± 1.9	10.9 ± 1.5	6–17	0.5
Azotemia (mg/dL)	42.2 ± 6.0	48.0 ± 17.6	21–59	0.7
Crea (mg/dL)	0.8 ± 0.2	1.4 ± 1.0	0.5–1.5	0.7
ALT (U/L)	41.9 ± 11.8	35.1 ± 8.3	21–102	0.1
AST (U/L)	34.6 ± 10.2	34.3 ± 12.9	23–66	0.9
ALP (U/L)	111.8 ± 28.2	110.3 ± 32.1	20–156	0.9
BIL (mg/dL)	0.3 ± 0.1	0.3 ± 0.1	0.1–0.5	1
GGT (U/L)	3.0 ± 1.4	2.4 ± 1.2	1.2–6.4	0.4
GLU (mg/dL)	73.8 ± 12.0	74.1 ± 13.5	65–118	0.9
TRIG (mg/dL)	80.0 ± 23.0	86.1 ± 22.3	20–112	0.5
CHOL (mg/dL)	193.6 ± 49.3	223.7 ± 63.8	135–270	0.3
HDL (mg/dL)	89.7 ± 19.1	47.4 ± 26	49–165	<0.0001
LDL (mg/dL)	87.9 ± 49.5	159.1 ± 51.1	5–86	0.01
TP (g/dL)	6.2 ± 0.5	9.1 ± 1.7	5.4–7.1	0.0003
Alb	3.4 ± 0.4	2.9 ± 0.5	2.6–3.3 g/dL	0.02

RBC: red blood cells; Hb: hemoglobin; Hct: hematocrit; MCV: mean corpuscular volume; MCH: mean corpuscular hemoglobin; MCHC: mean corpuscular hemoglobin concentration; Plt: platelet; WBC: white blood cells; Crea: creatinine; ALT: alanine aminotransferase; AST: aspartate aminotransferase; ALP: alkaline phosphatase; BIL: total bilirubin; GGT: gamma glutamil transferase; GLU: glucose; TRIG: triglycerides; CHOL: total cholesterol; HDL: high-density lipoprotein cholesterol; LDL: low density lipoprotein cholesterol; TP: total protein; Alb: albumin.

## Appendix A

### Appendix A.1. Ethical and Regulatory Approval

This study was approved by the Organism Proposed to Animal Welfare control (OPBA) at the investigators’ institution.

### Appendix A.2. Exosomes Isolation

Exosomes from all the blood samples collected (healthy and affected dogs) were treated with RNase A at 37 °C for 10 min (100 ng/ mL, Qiagen, Hilden, Germany) and then exosomes were isolated using a polymer-based kit, ExoQuick^®^ (System Biosciences, Palo Alto, CA, USA) according to the manufacturer’s protocol. Briefly, 250 µL of serum were centrifuged for 15 min at 3000× *g* to remove cell debris. The supernatant was transferred to a sterile tube and 63 µL of ExoQuick^®^ precipitation solution was added. After brief vortexing, the sample was incubated for 30 min at 4 °C, and then centrifuged at 1500× *g* for another 30 min at room temperature. After removing the supernatant, the pellet was re-suspended in 1/10 of the original volume using nuclease-free water.

### Appendix A.3. Characterization

After isolation, the serum exosome pellets were re-suspended in 1 mL of phosphate buffer solution (PBS) at pH 7.4 at a protein concentration of 1 µg/µL. Then 500 µL of the exosome suspension were labeled with 50 µL of 10× Exo-FITC for 10 min at 37 °C. The exosomes were then re-isolated, using an additional 100 µL of ExoQuick^®^ and incubated for 30 min on ice. Finally, the labeled exosome pellets were re-suspended in 500 µL of PBS and ready to be labeled with specific anti-canine antibodies on magnetic beads. In particular, anti- CD63-, CD9-, and CD81-coupled magnetic beads from SBI’s Exo-Flow IP^®^ kit (System Biosciences, Palo Alto, CA, USA), used at 1:3 dilution, were used for exosomal characterization as previously described [39]. Briefly, 50 µL of magnetic beads were incubated with 100 µL of the labeled exosomes overnight at 4 °C on an agitator. The following day, the beads/labeled exosomes were placed on a magnetic plate for 5 min, washed with 100 µL of 1× wash buffer once and analyzed by flow cytometry (FACSCalibur, BD Biosciences, San Jose, CA, USA).

### Appendix A.4. Analytical Methods

Exosomes were confirmed by TEM analysis and 5 µL of exosomes were resuspended in PBS containing 2% glutaraldehyde to fix them. The sample was applied to formvar 100-mesh grids and incubated for 10 min. Grids were washed twice with filtered distilled water and stained using 1.5% UA in water for 10 min; after that, they were washed with water to remove the excess staining solution and grids were air-dried. Images were acquired from grids using a FEI Tecnai 12 transmission electron microscope (FEI Company, Hillsboro, OR, USA) equipped with a Veleta CCD digital camera (Olympus Soft Imaging Solutions GmbH, Münster, Germany) and operating at 120 kV. Images were collected at magnifications of 30,000×, 49,000×, and 120,000×.

Dynamic light scattering and zeta potential determinations were performed with a Nano ZS 90 (Malvern Instruments, Orsay, France), which allowed the analysis of particles with sizes ranging from 10 nm to 3 µm. Exosomes derived from canine serum were diluted in 1 mL of PBS and zeta potential (electronegativity) and size distribution were analyzed at 37 °C according to the manufacturer’s instructions.

### Appendix A.5. Exosomal Protein Isolation and Quantification

The blood of ten affected and ten healthy dogs was used for this experiment. Purified exosomes were treated with 200 µL of lysis buffer (1% Triton X-100, 0.1% SDS, 0.1 M Tris HCl, pH 7) and protease inhibitors (Sigma-Aldrich, St. Louis, MO, USA). Protein concentration was determined by Bradford microassay (Bio-Rad Laboratories, Hercules, CA, USA) using BSA as standard and normalized to the starting amount of serum.

### Appendix A.6. miRNAs Isolation and miR-122-5p and RNU6 Analysis

Exosomes isolated from the serum of ten healthy and ten affected dogs were processed for miRNA isolation using a commercially available exoRNeasy^®^ Serum Plasma Kit (Qiagen) following the manufacturer’s instructions. In the lysis buffer were added 1 × 10^8^ copies of synthetic *Caenorhabditis elegans* microRNA (miRNA) oligoribonucleotides. Purified miRNAs were reverse transcribed using the miRCURY LNA^TM^ Universal cDNA synthesis kit II (Exiqon, Vedbaek, Denmark). Briefly, 50 ng of total RNA was combined with 2 μL of 5× reaction buffer, 1 μL of enzyme mix, and nuclease-free water up to a final volume of 10 μL. The reverse transcriptase PCR (RT-PCR) reaction was set as follows: incubation at 42 °C for 1 h, heat inactivation at 95 °C for 5 min, and immediately cooled to 4 °C using a thermocycler GeneAmp^®^ PCR System 2700 (Applied Biosystems, Foster City, CA, USA).

To verify the reliability of the developing protocol, one hundredth of the cDNA of six healthy dogs was amplified by quantitative RT-PCR (qRT-PCR). SYBR^TM^ Green master mix 2X (Promega, Madison, WI, USA) and miRCURY LNA^TM^ RNU6 reference gene specific PCR primer set (Exiqon, Vedbaek, Denmark) or miRCURY LNA^TM^ hsa-miR-122-5p specific PCR primer set (Exiqon) were used following the manufacturers’ instructions. Reactions were performed in triplicate and in a total volume of 20 µL. qRT-PCR reactions were performed using a BioRad iQ^TM^ 5 apparatus (Bio-Rad Laboratories) with the following conditions: initial denaturation step at 95 °C for 10 min, followed by 40 cycles of 10 s at 95 °C, and 1 min at 60 °C. Specificity of PCR products was checked by melting curve analysis. The same protocol was adopted to analyze the miR-122-5p expression profile of the remaining four healthy and ten diseased dogs. Cel-miR-39 was used to normalize between samples in the analysis. The results were analyzed using the comparative C_T_ (cycle threshold) method and the relative mRNA expression of miR-122-5p was determined using the C_T_ method comparing healthy and affected dogs.
